# Editorial: Lymphocyte functional crosstalk and regulation, volume II

**DOI:** 10.3389/fimmu.2023.1214843

**Published:** 2023-05-17

**Authors:** Raghvendra M. Srivastava, Menaka Thounaojam, Francesco M. Marincola, Anil Shanker

**Affiliations:** ^1^ Center for Immunotherapy and Precision Immuno-Oncology, Cleveland Clinic, Cleveland, OH, United States; ^2^ Lerner Research Institute, Cleveland Clinic, Cleveland, OH, United States; ^3^ Cleveland Clinic Lerner College of Medicine, Case Western Reserve University, Cleveland, OH, United States; ^4^ Department of Ophthalmology, Augusta University, Augusta, GA, United States; ^5^ Sonata Therapeutics, Half Moon Bay, CA, United States; ^6^ Department of Biochemistry, Cancer Biology, Neuroscience and Pharmacology, School of Medicine, Meharry Medical College, Nashville, TN, United States; ^7^ Host-Tumor Interactions Research Program, Vanderbilt-Ingram Comprehensive Cancer Center, Vanderbilt University School of Medicine, Nashville, TN, United States; ^8^ Vanderbilt Center for Immunobiology, Vanderbilt University School of Medicine, Nashville, TN, United States; ^9^ Vanderbilt Institute for Infection, Immunology and Inflammation, Vanderbilt University School of Medicine, Nashville, TN, United States

**Keywords:** NK cells, NKT cells, dendritic cells, immunotherapy, cancer, infections, tumor microenvironment, T cells

The immune system consists of specialized cells to perform specific activities to protect the body against various insults including cancer and infections (1). Immune cells often cooperate to mount effective immune responses (2). However, immune responses can be diminished by escape mechanisms employed by ever-evolving tumor cells and pathogens. Escape mechanisms often lead to acquired resistance to therapies (3–6). Crosstalk among a heterogeneous group of immune cells determines the therapeutic outcomes of immunotherapies.

Bulk RNA, single-cell RNA, and targeted next-generation sequencing technologies and computational approaches have greatly improved our understanding of immune subsets and their functional trajectories during disease progression (7, 8). Moreover, these technologies are advancing translational research and clinical applications. In this editorial, we highlight the findings of six articles featured in “*Lymphocyte Functional Crosstalk and Regulation: Volume II*”. As with Volume I (9), articles in Volume II provide novel insights into the interactions among immune cells in specialized microenvironments and emphasize the importance of considering these unique interactions when developing immunotherapeutic strategies (**Figures 1A–F**) (Wu et al., Zhu et al., Zhao and Yang, Du et al., Tischer-Zimmermann et al., Seliger and Massa).

**Figure 1:**
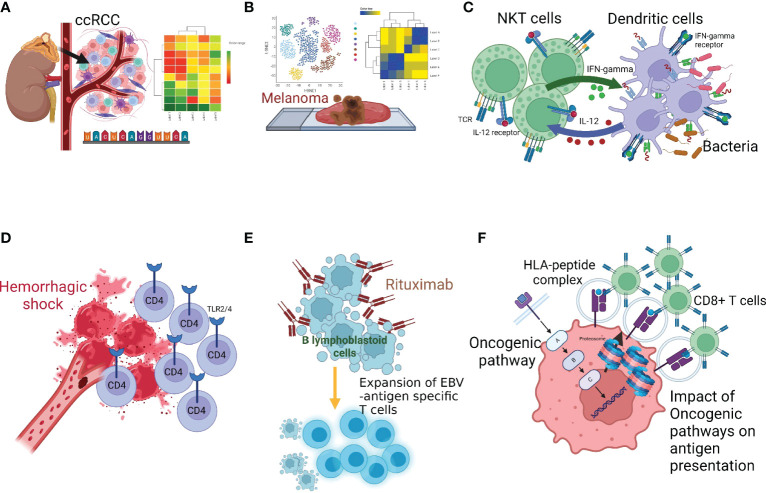
Multiple facets of innate-adaptive immune crosstalk. **(A)** Wu et al. reported genomics-based analysis of ccRCC tumor microenvironment and highlighted the importance of considering the immune-tumor crosstalk in clinical decision-making, **(B)** Zhu et al. performed genomics analysis in melanona tumor microenvironment and identified unique crosstalk between the tumor intermediate and CD8^+^T exhausted states, **(C)** Zhao and Yang described the bi-directional and reciprocal interactions between NKT and DC during bacterial infection, **(D)** Du et al. showed the role of Toll-like receptors (TLRs) and the tumor necrosis factor-α-induced protein 8-like-2 in CD4^+^T-cell activation following hemorrhagic shock, **(E)** Tischer-Zimmermann et al. demonstrates a novel mechanism of action of Rituximab to eliminate residual malignant cells by potentiating viral antigen cross-presentation to promote EBV-specific memory CD4^+^T-cell responses with high cytotoxic capacity, **(F)** Seliger and Massa reviewed how activation of oncogenic pathways and inactivation of tumor suppressor genes produces tumor-promoting hypoxic tumor microenvironment that leads to cancer immune escape.

Clear cell Renal Cell Carcinoma (ccRCC) is a heterogeneous, aggressive cancer representing ~70% of all RCCs (10, 11). It is difficult to predict the outcome of extensively used ICB treatment in ccRCC based on known immune molecular biomarkers (12). Few genomics-based studies have explored immunologic mechanisms at the tumor-immune interface in ccRCC (13). Wu et al. selected an 84-gene panel in ccRCC patients and classified them by using the CNMF algorithm in two distinct ccRCC molecular clusters, C1 (N=176) and C2 (N=333) in TCGA data. Investigators discovered how immune escape pathways disrupt the positive effect of immune cell infiltration in ‘cluster 1’ but not in ‘cluster 2’. Their findings emphasize the genomics-based evaluation of immune-tumor crosstalk during patient selection strategies (12).

Spatial distribution and functional state heterogeneity of CD8^+^T-cells in the tumor microenvironment (TME) may determine the clinical outcome of immunotherapies (14). In melanoma, Zhu et al. investigated the relationship between tumor cell states, CD8^+^T-cell exhaustion, and overall clinical outcomes. They utilized publicly available SCRNA datasets in melanoma and dissected the functional/exhaustion state of T-cells and receptor-ligand interaction between tumor cells and CD8^+^T-cells. Interestingly, they identified a unique crosstalk between the tumor intermediate state and exhausted CD8^+^T-cells. Tumor intermediate state and stemness are considered critical obstacles in immunotherapeutic approaches; therefore, these findings may be considered to counteract the development of immune resistance (15).

NKT are a unique subset of T-cells that recognize glycolipid antigens. The reciprocal interaction between DC:NK (16) and DC:NKT clusters is of great interest since NKT can produce a diverse range of cytokines (17). Zhao and Yang reviewed reciprocal interactions between NKT and DC to facilitate the adaptive immune response against infections. It is known that TCR-expressing NKT-cells share features of NK and T-cells (18). Cytokines secreted from NKT can induce DC maturation and facilitate T-cell activation. The review evaluates NKT subsets – NKT1, NKT2, NKT10, and NKT17 – regarding their differential ability to produce cytokines, occurrence in specific tissues, and transcriptional profiles. In addition, the unique characteristics of functional NKT subsets and their lipid ligands that cause NKT activation are discussed.

Excessive bleeding often leads to hemorrhagic shock, immune dysfunction, multi-organ failure, and death in extreme cases (19). Du et al. examined the role of Toll-like receptors (TLRs) and tumor necrosis factor-α-induced protein 8-like-2 (TIPE2) in the immune response to trauma. Their study shows that the expression of TIPE2, a negative regulator, is upregulated in CD4^+^T-cells after hemorrhagic shock. This upregulation is mediated by TLR2/TLR4 signaling and contributes to CD4^+^T-cell activation. Authors suggest that the TIPE2-TLR2/4 axis could be targeted to improve hemorrhage–associated multi-organ failures.

FDA-approved anti-CD20 mAb, Rituximab, is used to treat CD20^+^B-cell malignancies. Several mechanisms of action for Rituximab are known, such as ADCC and complement-mediated lysis. However, how long-term immune protection is induced by Rituximab and mechanisms of generation of Epstein-Barr Virus (EBV)-specific T-cells remain unclear (20). Tischer-Zimmermann et al. explored how rituximab treatment leads to the generation of EBV-specific memory T-cells. The findings suggest that Rituximab eliminates residual malignant cells by potentiating viral antigen cross-presentation to promote EBV-specific memory T-cell responses with high cytotoxic capacity.

An unproductive cancer antigen processing and presentation remains a dominant mechanism of immune escape (3, 21–23). Seliger and Massa highlighted how activation of oncogenic pathways and inactivation of tumor suppressor genes produces hypoxic tumor-promoting TME. Authors described several detrimental consequences of constitutively activated tumor intrinsic oncogenic pathways (*K-RAS* mutation, TSG liver kinase B mutation, WNT-β-catenin pathway, *myc* oncogene amplification, overexpression of HER-2/EGFR genes) on a variety of immune cells. Similarly, the impact of a loss of the tumor-suppressor gene, *PTEN*, on T-cell infiltration and Treg frequency was discussed (24). The immunological consequences and role of ten-eleven translocation (TET) family mutations in hematologic malignancy, IDH-1/2 mutation in Glioma (25), inactivation of von Hippel Lindau (VHL) in RCC are highlighted (26). Moreover, the immunoregulatory role of tumor growth receptors, the evolution of genomic alteration, secreted cytokines/chemokines in the context of specific cancer, and their genetic determinants are discussed. An in-depth investigation is warranted on how these pathways operate when tumor-targeting drugs and immunotherapies are applied together and how tumor cells readjust to display therapeutic or acquired resistance.

In conclusion, the articles published in this Research Topic (Wu et al., Zhu et al., Zhao and Yang, Du et al., Tischer-Zimmermann et al., Seliger and Massa) provide critical insights into immunological crosstalk within their specialized microenvironments (27), advancing the knowledge of the elements and mechanisms that underlie innate-adaptive immune interactions (2, 9) in various pathological conditions including TME (27–34). These studies shed light on critical cellular players and molecular mechanisms that regulate immunological crosstalk and impact various pathologic conditions, including hemorrhagic shock, cancer, and infectious diseases. The findings underscore the importance of considering the complexity of immune subsets and their microenvironments when developing therapeutic strategies. The development of various technologies, such as single-cell and spatial transcriptomics, has begun to uncover the complexities of specialized microenvironments and how treatments facilitate immune effector crosstalk. Further research and technological advancement to tease the underpinnings of immunological crosstalk will contribute to developing more effective and long-lasting treatments for various immune-related pathologies.

## Author contributions

RS, MT and AS conceived, designed and wrote the manuscript. FM provided intellectual review and feedback. All authors read and approved the final manuscript for publication.
